# Supplementary material for: Past and present goals are represented concurrently during visual search
Source: PLoS Biol. 2026 May 4;24(5):e3003779. doi: 10.1371/journal.pbio.3003779 (PMC13155657; doi:10.1371/journal.pbio.3003779)
Supplement: Attachment — Submitted filename: response_letter.docx [file pbio.3003779.s011.docx]

Dear Dr. Schnell and other editorial staff,

We thank the reviewers for the high-quality comments on our manuscript. Accommodating the comments has greatly improved our manuscript by more directly ruling out eye movements as a potential alternative explanation to our results, and to further examine *when* previous targets are decodable. We have replied to each comment point-by-point in blue, and provide the corresponding changes to the manuscript in green.

On behalf of all authors,

Kind regards,

Damian Koevoet

**Point-by-point replies**:

Reviewer #1: The present work by Koevoet and colleagues reports reanalyzed EEG data from two earlier publications, now with a focus on how previous-trial target locations affect visual search, and how it interacts with current-trial target location. Using elegant IEM and RSA analyses, they observe that previous-trial target locations get consistently reactivated during the next stimulus event (the next search array, as well as a cuing display) and this signal coexists with the current-trial target information. Overall, I thought that the manuscript was interesting, well-done and that it will make a substantial contribution to the literature. Yet, I also had a couple of concerns and questions, which I hope the authors can address.

We were happy to read the reviewer’s overall positive assessment of the manuscript.

Major
1) I appreciated the analysis of the eye-movement data for experiment 1, which yielded clear results. Yet, there are a couple of other things that I think deserve attention in this regard: a) no such results are provided for experiment 2, which seems like an oversight; b) while reassuring concerning eye movements with regard to the previous target location, participants seem to quite clearly move their eyes in general (but see also last minor point), and likely towards the current target location (which is hard, if not impossible, to fully avoid). I think it would also be important to report results with regard to the eye-movements towards the current target location. c) related to the previous points, I was surprised that I did not see any information in the method section about how eye-related activity may have been attenuated, artifact rejection aside. Why not use ICA to remove as much eye-related activity as possible from all EEG channels, in order to avoid a direct influence in the multivariate results? If I am not overlooking something, I feel that it would be important to still do that.

We agree with the reviewer that it is important to consider potential effects of eye movements on our decoding results. In addition to our EOG analyses from Experiment 1, we conducted multiple additional analyses. As suggested by the reviewer, we considered an ICA approach to remove ocular artifacts. However, given that we had access to eye-tracking data, this allowed us to take a more direct approach. Rather than relying on ICA, which removes the ocular dipole artifacts but does not account for the downstream consequences of (micro)saccades on the EEG signal, we instead used the gaze data itself to directly examine what spatial information it contained and whether it could account for our decoding results.

Specifically for Experiment 2 (*n* = 41 out of 42 participants), we checked whether gaze tracked the previous and/or current target location (see figure below). As can be seen on the y axes, gaze deviations from the center of the screen were very minimal (<0.15 dva), indicating that our artifact rejection algorithm adequately cleaned the data from ocular artifacts. We found that in the covert attention condition (left side of the figure), gaze tracked the current target position (as reported in the original report, Foster et al. 2020; *Journal of Neuroscience*). Moreover, we found that gaze tracked the previous target position to some extent, but gaze pointed toward the opposite position: when the previous target was presented on the left, gaze tended to point more rightward and vice versa. However, this effect was not reliable for the up vs. down comparison. We note that this effect was very small in terms of dva as well as compared with the much stronger way in which gaze tracks the current target position. Thus, while gaze seems to track the previous target position in the covert condition, this effect was small in terms of dva, showed an opposite pattern as expected, and was not consistently present across directions.

In the neutral condition (right side of the figure), gaze did not reliably track the current, nor the previous target position. Therefore, gaze cannot fully explain our decoding results: neural activity reliably tracked the current and previous target positions (Figures 2 and 3) while gaze did not reliably track these positions.

We have added this analysis to the Supporting Information as follows:

Supporting Figure 4. Gaze analyses for the covert (left) and neutral (right) conditions of Experiment 2 (*n* = 41 out of 42 participants. One participant was excluded due to unusable eye-tracking data). In the covert condition, gaze reliably pointed toward the cued location. Gaze seemed to point away from the previous target location, but this was not reliable across direction comparisons. In the neutral condition, gaze did not reliably point toward the current nor the previous target location. This rules out the possibility that gaze fully accounts for the observed neural decoding results. Horizontal black lines indicate significant differences between direction conditions (*p* < .05).”

Nevertheless, to rule out the possible influence of eye movements on our decoding of the previous target position more directly, we conducted an additional analysis. For each position, separately for the covert and neutral condition, we classified trials as either biased toward or away from the previous target position. We split trials in a relative manner using a median split, that is we classified 50% of trials as biased toward the previous target position and the other half as biased away from the previous target position. This produced considerable gaze biases either toward or away from the previous target position.

We then repeated the IEM procedure as conducted in the main paper, but now we trained on the average of the biased toward and away trials. Training on the average EEG signal should cancel out the opposing influence of gaze on the neural signal between toward and away biasing trials. This approach limited the possibility that gaze biases influenced our decoding results as much as possible while still retaining a high number of trials (and thus statistical power). The analysis revealed that the previous target remained decodable across both toward and away biased trials, providing strong evidence that gaze did not drive our decoding results.

We have also added this analysis to the Supporting Information and refer to our eye-movement control analyses in the main paper:

Supporting Figure 5. Gaze bias control analysis for Experiment 2 (*n*=41 out of 42 participants). We created two bins of trials wherein gaze was either biased toward or away from the previous target location. Specifically, for each participant, condition and location separately, we median-split trials based on how close gaze was from the previous target location (averaged 500-1,250ms after cue onset). As can be seen in A and B, this created considerable gaze biases toward or away from the previous target location (pie plots correspond to previous target locations). Then, we reconducted the inverted encoding modeling procedure as in the main paper but we now trained on the average neural data across the toward and away trials. This way, opposing gaze biases should be effectively canceled out and can no longer affect the neural decoding results. C Our results demonstrate that previous target locations were reactivated upon cue onset, and later upon array onset. Crucially, this occurred in both toward and away biased trials, and we found no differences between these conditions. This rules out miniature gaze biases as a confound to our neural decoding results of the previous target location. Horizontal orange and pink lines indicates significant CTF slopes above 0 (*p* < .05). All error bars and bands reflect standard errors of the mean.”

“In a series of control analyses, we ruled out that eye movements drove the spatial selectivity in the reconstructed CTFs throughout the paper (Supporting Figures 3-5). These analyses showed that reactivation of previous target locations was not driven by eye movements but by neural activity.”

Together, careful gaze position analyses demonstrated that gaze cannot account for the decoding of the previous target position. Instead, we conclude that the previous target position is neurally reactivated.

2) One of the most frequent words in the present manuscript is "reactivated". I can in principle agree with its use, but I think it would then still be helpful to show that at some point the previous target location was deactivated. Would it be possible to show that preceding the n trial (target or cue, respectively for Exp1 and Exp2), the previous target location could no longer be decoded from the data? I believe that there were relatively long inter-trial intervals, which might be beneficial here. It may not be relevant, but some of the results struck me as showing very early effects of previous-target location (as early as 90 ms for the RSA of Exp1, but see also first minor point below), and I was wondering whether there was any way in which the baselining procedure in combination with the onset of a new stimulus may have temporarily suppressed an ongoing signal, rather than reactivating it. I am not necessarily assuming that this will be the case at all, but I would find it useful to show.

We agree that it is important to consider when previous targets are decodable from the EEG signal. Before adressing the comment with an additional analysis, we’d like to raise the fact that the timing of clusters is not a reliable estimate of ‘when’ a position becomes decodable. Instead cluster-based approaches specfically test whether there is an effect at all without precise temporal precision. Sassenhagen & Draschkow (2021; *Psychophysiology*) indeed showed in simulated data that cluster-based permutation tests can estimate onset latencies of effects earlier in time. Therefore, the 90ms onset latency has to be taken with a grain of salt.

We now describe this in the Methods:

“We note that identified significant clusters are not a reliable measure of when an effect precisely occurs, but we chose to report the cluster sizes as recommended by Sassenhagen and Draschkow [74].”

The reviewer suggests to consider when a target position is deactivated (i.e., no longer decodable) during the intertrial interval. We agree that this is an interesting analysis to conduct. Unfortunately, participants could blink during intertrial intervals, and these epochs are therefore very likely to be contaminated with ocular artifacts.

We therefore chose to tackle this issue using another analysis. In particular, we considered the possibility that prior targets were already decodable during the baseline period prior to search array onset. If prior targets are really reactivated due to stimulus onset, the prior target should not be electrically active before current display onset. If this were to be the case, it is possible that our baseline-correction may have caused the observed reactivations after stimulus onset. To circumvent this issue, we reconducted our IEM procedure but now when using the derivative of the voltage signal across time. In other words, the IEM is now trained on changes in voltage from sample to sample. This procedure does not require any baseline correction, and therefore completely avoids possible problems associated with baseline correction altogether.

In Experiment 1, we found that changes in voltage occuring ~200ms after stimulus onset revealed the position of the previous target. In Experiment 2, we observed reactivation of the previous target position in the covert and neutral conditions. In particular, we observed reactivation in the covert condition after cue onset, and another bump in reactivation was observed upon array onset. In the neutral condition, we only observed a significant reactivation upon search array onset (as in the RSA analysis). We also found that the reactivation in the neutral condition was stronger upon array onset than in the covert condition. This can be explained by considering the nature of the derivative data: based on the results reported in the main paper, it is likely that the prior target position remains activated within the cue-delay period in the covert condition, but if it is coded in a stable manner this would not be decodable using the derivative voltage signal. In other words, the previous target is likely already active in the EEG signal prior to array onset in the covert condition, and therefore the derivative response to the search array holds less information about the prior target position than in the neutral condition.

Together, these analyses demonstrate that changes in voltage occuring *after* stimulus onset carry information about the previous target position. This is strongly indicative of a reactivation, and is consistent with an activity-silent account of history-driven selection.

We have added this analysis to manuscript:

“One alternative possibility is that previous target locations were electrically active prior to search array onset, but appear as a transient reactivation due to the application of a baseline correction. We ruled out this possibility by calculating thederivative of voltage across time (i.e. changes in voltage from sample to sample), which does not require baseline correction, and reconducted the IEM procedure on this data (Supporting Figure 2). We found that voltage changes after stimulus onset revealed the previous target location. This is strongly indicative of a reactivation, and is consistent with an activity-silent account of history-driven selection.”

Supporting Figure 2. Inverted encoding modeling results using the derivative of the voltage signal (i.e. change in voltage from sample to sample). Importantly, this signal does not need any baseline correction, enabling us to examine if voltage changes after stimulus onset were sensitive to the previous target location. We found highly similar findings as those reported in the main paper for Experiment 1 (left) and 2 (right), ruling out the possibility that our previous target location decoding by the baseline correction procedure employed in the main paper. We also found that the reactivation in the neutral condition was stronger upon array onset than in the covert condition. This can be explained by considering the nature of the derivative data: based on the results reported in the main paper, it is likely that the prior target location remains activated within the cue-delay period in the covert condition, but if it is coded in a stable manner this would not be decodable using the derivative voltage signal. In other words, the previous target is likely already active in the EEG signal prior to array onset in the covert condition, and therefore the derivative response to the search array holds less information about the prior target location than in the neutral condition. Horizontal colored lines corresponding to conditions represent clusters where CTF slopes are reliably higher than 0 (*p* < .01). Horizontal black lines indicates significant differences between covert and neutral trials (*p* < .05).

3) I would appreciate it if the authors could check whether the behavioral results were also influenced by full repetitions (same target location and same target stimulus/response), which could inflate the target-distance effects, in line with event-coding theories etc. The easiest way to quickly explore this would be to limit the analysis to only target-alternation trials (where the required response on trial n is opposite from that of trial n-1). I think this would be entirely sufficient to report in the response letter, but since it is really easy to explore, I would like to see this data, even if some of the references in the intro might already say something to this effect in principle.

Indeed, event-coding theories would predict that the previous response and target position would be represented as a single ‘event’. We followed the reviewer’s suggestion and compared response times between full repetitions (motor and spatial position) partial repetitions (only motor, only position) and non-repetitions. However, our data did not reveal a very consistent picture.

In Experiment 1, we observed that responses became more accurate when either responses or positions were repeated across consecutive trials. However, if anything, accuracy seemed to slightly decrease when both the response and position matched across trials. This argues against the proposition that motor repetitions may have driven our priming effects. As for response speed, we found that responses were faster whenever positions matched across consecutive trials, and in easy trials there seemed to be a slight further increase in speed when both the position and response matched on consecutive trials.

In Experiment 2, we found that for accuracies, position only matches yielded the highest accuracies. Moreover, accuracies seemed lowest when both position and reponses matched on consecutive trials, arguing *against* event-coding (and indicates that spatial priming is not a motor confound). However, response times did favor an event-coding interpretation as responses were fastest in full match trials.

Together, we found no consistent evidence that our position priming effects were driven by repeated motor responses nor by a repeated ‘events’. While important to consider, event-coding was not the core focus of our paper and we therefore followed the reviewer’s recommendation of not adding these results to the manuscript.

4) This is more medium than major, but for the cue-based current-target location information, how can this be separated from simply information about the nature of the cue itself? It is also a spatially-selective stimulus, pointing in the relevant direction. Not a major concern, though, just a question for clarification.

The reviewer is right in that the presented cue itself in Experiment 2 contains spatial information. Although we cannot fully rule out that the stimulus itself contributed to our decoding results, we deem it unlikely this accounts for our conclusions for multiple reasons.

First, we want to note that in the inverted encoding modeling analyses, we exclusively decoded the target from the previous trial which always differed from the current target. Therefore, the spatial information of the cue was never informative regarding the past target position.

Second, participants responded faster and more accurately in the covert compared with the neutral condition (Figure 2), indicating that they had shifted attention to the relevant target position prior to search array onset.

Third, in recent work we used the same cues as those presented in Figure 2. Using these cues, we were able to detect differences in spatially selective neural activity between attentional conditions (Koevoet et al., 2026; *Journal of Neuroscience*). This implies that the sensory information contained within the cue stimulus is not driving the full decoding results. Moreover, the presented cues were small in size (0.125° x 0.08°), and in 26/42 participants the relevant cue direction was closely luminance matched with the other lines representing other possible directions. We therefore think it unlikely that cue stimuli carrying such little sensory energy are able to evoke signals that allow for the decoding of the target position throughout the delay period Instead, our decoding of the current target location prior to search array onset in Experiment 2 is likely the combination of transient sensory information contained within the cue as well as an attentional effect that is sustained throughout the delay period.

We have added these considerations to the manuscript, as this is also important to consider when comparing the representational strength of the current versus the past target position.

“One potential limitation of the decoding of the current target location in the covert condition, is that the cue stimulus itself carried spatial information. However, we believe that the specific properties of the cue cannot account for the sustained decoding of the current target location for multiple reasons. First, participants responded faster and more accurate in covert compared with neutral trials, indicating that attention was deployed prior to search array onset. Second, cue stimuli were small in size (0.125°x 0.08°) making it unlikely that their weak sensory energy allowed for sustained decoding throughout the delay period. Third, recent work using the same cue stimuli demonstrated differences in spatial selectivity between preparatory overt and covert shifts of attention [37], indicating that the neural decoders are picking up on attentional processes, likely alongside transient spatially selective sensory input provided by the cue stimulus. These arguments support the conclusion that decoding of the current target location decoding reflects the deployment of covert spatial attention.”

5) Would it be possible to report information on which EEG channels contributed most to the current results, or in any other way add a spatial dimension to the results?

We have added a searchlight analysis to uncover which EEG channels were most important in representing the prior target position. In Experiment 1, only 20 electrodes were used, located primarily in posterior areas, making inferences about spatial dimension difficult. We therefore only conducted this analysis for the data from Experiment 2, as here data was recorded using 30 electrodes distributed more evenly across the scalp.

In the searchlight analysis (based on Koevoet et al., 2026; *Journal of Neuroscience*), we conducted our IEM analyses as reported in the main paper. However, we now iteratively selected groups of five neighbouring electrodes across the scalp and calculated CTF slopes. Then, we determined a channel’s spatial selectivity to the previous target by averaging the CTF slopes of each electrode group the channel was included in. This allowed us to visualize spatial selectivity to the previous target position across electrodes, and collapsed across all eight possible positions. To ease visualization, we averaged spatial selectivity for each electrode from array onset until the end of the epoch (700-1250ms after cue onset). As can be seen below, the previous target position was primarily represented in posterior electrodes in both covert and neutral trials.

Since the topographies were very similar between conditions, we have updated Figure 2 to now include the averaged topography of both conditions:

Figure 2: Task and results from Experiment 2. A Cued visual selection task. Participants searched for the gap in the target diamond in covert and neutral conditions. Dimensions do not match those in the experiment but have been changed here for clarity. B Response times separately for the covert (red) and neutral (gray) conditions on the left. Response times are plotted as a function of distance between the current and previous target location. On the right: slopes (i.e. beta) calculated using linear regressions per participant to assess the spatial gradient of intertrial priming. Black dots represent individual participants. C Same as for B but for accuracy data. D CTF slopes (i.e. spatial selectivity) from cue onset over time for the covert (red) and neutral (gray) conditions. Topography reflects the outcome of the searchlight analysis averaged across covert and neutral conditions, where higher values indicate that an electrode elicited a steeper CTF slope. Horizontal red and gray lines represent significant clusters (i.e. CTF slopes above 0; p < .05) for covert and neutral conditions, respectively. Horizontal black line represents a significant cluster-corrected difference between conditions (p < .05). All error bars and bands reflect standard errors of the mean. RT = response time. *p < .05, **p < .01, ***p < .001.

We also describe the searchlight analysis in the paper:

“To further characterize the reactivation pattern, we conducted a searchlight analysis [53] to visualize the contribution of each electrode across the scalp to our IEM results [37]. To this end, we iteratively selected groups of four or five neighbouring electrodes across the scalp and reconducted the IEM procedure. We calculated an electrode’s spatial selectivity to the previous target location by averaging across all electrode groups that an electrode was a part of. This way, we were able to visualize spatial selectivity for each electrode while collapsing across all eight target locations. To facilitate visualization, we averaged CTF slopes upon array onset (700-1,250 ms after cue onset), and as we found highly comparable results for the covert and neutral conditions we averaged topographies across conditions. Closely resembling topographies tracking current target locations [54, 55], we found that especially posterior channels tracked the location of the previous target location.”

Minor
1) I was a bit surprised by the 6-Hz low-pass filter. This is probably fine, even if somewhat at odds with the highly-resolved testing logic, but should probably at least be acknowledged concerning timing accuracy (assuming a non-causal filter, e.g. onset latencies will shift forward, right?).

We based our 6Hz low-pass filter on Bae & Luck (2018; *Psychological Science*), wherein they demonstrated that previous memorized orientations were reactivated during the current working memory trial. The 6Hz filter is not specific to IEMs, and in principle IEMs could be trained on any multivariate signal. We here chose to apply the low-pass filter because it reduced noise in our decoding results.

Specifically, we applied a zero-phase FIR filter using MNE-Python, which applies the filter both forward and backward to eliminate phase shift. Therefore, the low-pass filter should not have shifted onset latencies forward. We tested this quantitively by applying a 30Hz low-pass filter to our data from Experiment 1 and reconducting the IEM analysis. In the figure below, we present our results from the main paper on the left, and the results using the 30Hz low-pass filter on the right. Although the overall CTF slopes are noisier across time, we found that in both the easy and hard conditions previous target positions were decodable. Critically, the timing of these effects was highly similar regardless of whether we applied a 6Hz or 30Hz low-pass filter. Therefore, the employed 6Hz filter did not make reactivations appear earlier in time.

Although we have chosen to not include this analysis in the paper for brevity, We have elaborated on the filter in the Methods section as follows:

“We then applied a 6 Hz lowpass filter [as in 43]. Specifically, we used a zero-phase FIR filter, which applies the filter both forward and backward to eliminate phase shift, and consequently does not shift the data across time (using *filter_data* from the *mne* package). These baseline-corrected and filtered data served as the input to the decoding analyses.”

2) Readers that are not steeped in the technique (myself included) may think that the differences in semi-partial correlation between current and previous target location are large to an extent that feels incomparable; this may be worth contextualizing.

The reviewer is correct that differences in semi-partial correlation between current and previous targets are large. In our case, the semi-partial correlations are akin to standardized beta values obtained from linear regression (although we here do not assume linearity and therefore opted for rank-correlation), which represent the standardized effect of a predictor. Therefore, the semi-partial correlation values are directly comparable, and it is clear that current targets explain much more variance in the ongoing EEG signal than previous targets.

We now elaborate on the RSA approach and the semi-partial correlations in more detail:

“Equipped with both the theoretical (i.e., predictors) and empirical (i.e., outcome) RDMs, we were able to test whether previous and current target positions were represented in the neural data. As we did not assume any linear relationships, we used semi-partial rank correlations [62, 60, 52]. Semi-partial rank correlations isolate the relationship between a predictor and the outcome while controlling for the influence of another predictor. In our case, this allowed us to isolate the relationship between the location of the previous (current) target and the neural data while accounting for the influence of the current (previous) target location. The obtained semi-partial rank correlations are a standardized measure of the strength of the influence of a predictor on an outcome variable, and therefore we were able to compare how strongly current and previous target locations were neurally represented. We repeated the full RSA procedure across all time bins.”

3) I am wondering whether the authors have any idea for why the HEOG in Fig. S1 is just a moderate drift, whereas the VEOG shows a large-amplitude, and highly temporally-structured response, with a very early negative peak (already around 100 ms, which seems very fast). Are EOGs expressed as bipolar montages, or with regard to the mastoid reference? From the looks of it, it seems like the HEOG might be from a bipolar montage, and the VEOG from a mastoid reference, but I am just speculating. 

The reviewer is correct that the HEOG is expressed as a bipolar montage while VEOG was recorded from a single electrode (placed below the right eye) with a mastoid reference. This could indeed explain the difference between HEOG and VEOG responses across time. Nevertheless, even if the VEOG was recorded using a mastoid reference, the difference between up and downward previous target positions remains relevant to examining whether gaze tracked the prior target position. We have now added this to the caption of the figure (now Supporting Figure 3):

Supporting Figure 3. Eye-movement control analysis for Experiment 1. To rule out eye movements as a possible confound for the decoding analysis of the previous target location reported in the main paper, we grouped trials in bins of two prior target locations left, right, up or down. We first baseline corrected HEOG and VEOG data using the median voltage 250 ms prior to array onset. We then compared HEOG between trials where the prior target locations was on the left or on the right. We then compared VEOG between trials where the prior target locations was up- or downward. Neither of these analyses showed evidence that the prior target location was tracked by eye movements. HEOG (left) is plotted separated by trials where the prior target location was on the left or on the right. VEOG (right) is plotted separated by trials where the prior target location was up- or downward. Note that while HEOG was recorded using a bipolar montage (i.e., an electrode placed at the outer canthi of each eye), VEOG was recorded with a single electrode with a mastoid reference. This likely accounts for the different HEOG and VEOG responses across time. Nonetheless, comparisons between right and left, as well as up and down are still relevant to examine whether gaze tracked the previous target location. Error bands reflect standard errors of the mean.

Reviewer #2: In this manuscript, the authors investigated the interplay of goal-driven and history-driven influences on visual search. Using EEG decoding and representational similarity analysis (RSA), the authors demonstrate across two tasks that prior target locations are neurally reactivated even when task-irrelevant, and that current and previous targets are concurrently represented during visual search. This was consistent with the behavioral results which demonstrated intertrial priming effects that incorporated localized attentional interference. There are a number of novel findings presented in the work and I think it makes an important contribution to attention research, particularly those who study visual search and selection history. I think the research was well designed, methodologically rigorous, and the conclusions follow logically from the findings. As such, I only have some queries for the authors and one minor recommendation for revision. To be clear, I don't feel that the revision is strictly necessary, in my view there are no significant issues with the work, but I have recommended minor revisions here so that the authors have an opportunity to incorporate this change if they choose to.

We thank the reviewer for their positive assessment of our work, and we appreciate the reviewer’s approach.

Minor revision:
In general I found the discussion to be quite cursory. There are some interesting ideas here, particularly the "operate-to-reactivate" section but they are explored only briefly and don't read as if they have been thoroughly considered. 

In particular, one of the most interesting parts of the results to me was the fact that the reactivation of prior targets was lower in the neutral cue condition than the covert cue condition. The introduction of a goal-driven attentional influence in the covert condition was framed as something that, if anything, should decrease the reactivation of prior targets. The results seem to show the reverse. Relative to conditions with a neutral cue, covert spatial attention toward a new target location increased the reactivation of prior targets. This is seen numerically in the IEMs (and briefly statistically significantly following the array presentation in Figure 2D), but is also supported in the RSA analysis where the previous target location was not significantly reactivated in the neutral condition. While the conclusions seem to be suggesting that prior target representations are reactivated "despite covert spatial attention," these results could also be used to suggest that prior target reactivation in this task was enhanced by it. Aside from a sentence relating this to the "operate-to-reactivate" idea, this pattern of results isn't really discussed.

That is the main example of a discussion point that I felt went under-explored, but I do feel that this applies more generally in the discussion of the paper. Ideas are introduced very briefly and any evidence to support them is also brief. Some prior work is cited to support some of the ideas, but isn't really explained or discussed in a detailed way.

We appreciate the reviewer’s detailed comment regarding the discussion section.

Regarding the more specific point. We see the reviewer’s point that we mainly write about that prior targets were reactivated “despite covert spatial attention”. We wrote the paper this way because we expected the reactivation of previous target positions to be attenuated (as we found in the behavioral results) prior to conducting the analyses. However, we do agree that in the discussion this idea went under-explored. We therefore have extended the operate-to-reactive paragraphs to provide more detail and to make a few more specific suggestions.

“While our findings possibly demonstrate that task-relevant search arrays can serve as "ping" stimuli to reveal latent activity-silent states [45, 39, 23], the current results also point to an alternative explanation: history-driven signals may emerge through operational engagement of the priority map rather than through sensory perturbation per se. Instead of requiring external sensory perturbation to access latent activity-silent states, history-driven representations may be automatically co-activated whenever the priority map is updated or recruited for search initiation. Several aspects of our data support this "operate-to-reactivate" account. Most notably, informative cues reliably evoked spatially selective neural activity to prior target locations (Figure 2), despite their minimal sensory energy—arguably insufficient to act as ping stimuli. Such reactivations may have emerged because the priority map was engaged during covert trials upon cue onset, which also accounts for the (somewhat) stronger reactivations compared with neutral trials. Moreover, previous target reactivation onset occurred earlier in covert versus neutral conditions, where priority map engagement likely occurs sooner following array presentation. Similarly, this accounts for the differential reactivation latencies between easy and hard conditions (Figure 1C): if participants engage the priority map (i.e. by updating the goal-driven layer) earlier in easy trials where targets "pop out", history-driven reactivations should emerge correspondingly earlier compared with hard conditions.

The operate-to-reactivate framework suggests that the priority map has an integrated representational structure [4, 69, 70], where engaging one layer of the map reveals information embedded in other layers. We here provide the first tentative evidence for this by showing that engagement of the goal-driven layer reactivated latent history-driven information. If the priority map indeed has an integrated representational structure, activation of the stimulus-driven layer should also reveal history-driven information, which accounts for previous results obtained with pinging paradigms [39, 23]. Notably however, recent work demonstrated that goal-driven attention can be engaged during the presentation of pinging displays [71], which could have inadvertently activated the integrated priority map rather than merely probing latent states through sensory perturbation [see 23]. While further research is needed to establish the boundary conditions for history-driven reactivations, our findings demonstrate that goal- and history-driven influences are represented simultaneously and distinctly in the brain, regardless of the precise underlying mechanism.”

General queries: 
1.      I was interested to see that the authors applied a 6 Hz low-pass filter during preprocessing. I'm curious as to the rationale for this threshold as it seems much lower than anything I would usually expect to see. Is this something specific to IEMs?

We based our 6Hz low-pass filter on Bae & Luck (2018; *Psychological Science*), wherein they demonstrated that previous memorized orientations were reactivated during the current working memory trial. The 6Hz filter is not specific to IEMs, and in principle IEMs could be trained on any multivariate signal. We here chose to apply the low-pass filter because it reduced noise in our decoding results.

Specifically, we applied a zero-phase FIR filter using MNE-Python, which applies the filter both forward and backward to eliminate phase shift. Therefore, the low-pass filter should not have shifted onset latencies forward. We tested this quantitively by applying a 30Hz low-pass filter to our data from Experiment 1 and reconducting the IEM analysis. In the figure below, we present our results from the main paper on the left, and the results using the 30Hz low-pass filter on the right. Although the overall CTF slopes are noisier across time, we found that in both the easy and hard conditions previous target positions were decodable. Critically, the timing of these effects was highly similar regardless of whether we applied a 6Hz or 30Hz low-pass filter. Therefore, the employed 6Hz filter did not make reactivations appear earlier in time.

Although we have chosen to not include this analysis in the paper for brevity, We have elaborated on the filter in the Methods section as follows:

“We then applied a 6 Hz lowpass filter [as in 43]. Specifically, we used a zero-phase FIR filter, which applies the filter both forward and backward to eliminate phase shift, and consequently does not shift the data across time (using *filter_data* from the *mne* package). These baseline-corrected and filtered data served as the input to the decoding analyses.”

2.      In the IEMs, would adjacent target locations be more likely to be classified as one another? In these analyses you removed trials where target location had been repeated, to avoid having your "reactivation" driven by trials where the model simply decodes the current target location. However, if adjacent target locations are more similar than distant target locations, then doesn't this imply your effects could be driven by trials where the current target is adjacent to the previous target? The effect wouldn't be as strong as for two targets in the exact same location, but could this still produce an above chance effect?

Indeed, we did not include trials wherein the current and past positions were the same to avoid ‘spillover’ from the current position to our past position decoding. In the manuscript, we addressed this possibility more directly by using RSA – which considers the position of both the past and current target position simultaneously. However, based on this comment we also examined the IEM analyses in more detail.

To further rule out the possibility of a ‘spillover’ effect from the target position, we changed the basis functions for our inverted encoding modeling procedure. Specifically, we used stickfunctions, which indicate that a channel response should only be present for the prior target position, and there should be no activity channel responses to other positions. Because current and previous target positions were never the same in our IEM analyses, these basis channel responses would rule out the possibility that the position of the current target position contributed to prior target position decoding. Moreover, this approach allowed us to rule out that the spatial tuning to the previous target position was driven by our choice of basis channel function instead of ‘true’ spatially tuned activity.

Results from the stick function analyses were overall highly similar to our data from the main paper. We observed reliable spatial selectivity to the previous target position in all conditions. This analysis suggests that ‘spillover’ from the current target position cannot account for spatially selective signals tuned to the prior target position as revealed by the IEM analyses.

We have added this analysis in the Supplementary Materials:

Supporting Figure 1. Although we ensured that previous and current target locations never matched in the inverted encoding modeling (IEM) analyses, we here conducted an additional control analysis to ensure current target locations did not influence our previous target location decoding results. To this end, we reconducted the IEM analyses using different basis functions wherein we did not assume a spatially-tuned profile but instead functions that predicted a response at the previous target location, and no response at all other locations. We found highly similar findings as those reported in the main paper for Experiment 1 (left) and 2 (right), ruling out the possibility that our previous target location decoding was driven by the location of the current target. Horizontal colored lines corresponding to conditions represent clusters where CTF slopes are reliably higher than 0 (*p* < .05). Horizontal black lines indicates significant differences between covert and neutral trials (*p* < .05).

I understand that this doesn't explain all of your results, so your main conclusions likely stand, but might it explain why target representations disappear in the RSA analysis for the neural cue condition (where, to my understanding, this problem would be accounted for)?

The reviewer is right in that the RSA analysis accounts for this possibility altogether. Here, the previous target position explains *distinct variance* in the EEG signal that cannot be accounted for by the current target position.

Overall, I think this is a well-executed study that adds some innovative methods to the field and several novel findings that advance our understanding of attention. As mentioned, I found relatively little that I feel needs revising and would leave it to the authors discretion. The study appears to be methodologically rigorous, and of interest to researchers in cognitive neuroscience and related disciplines

Again, we are happy to read the reviewer’s positive assessment of our manuscript.
